# Hybrid Additive and Subtractive Manufacturing Method Using Pulsed Arc Plasma

**DOI:** 10.3390/ma16134561

**Published:** 2023-06-24

**Authors:** Xiaoming Duan, Ruirui Cui, Haiou Yang, Xiaodong Yang

**Affiliations:** 1 Department of Mechanical Engineering and Automation, Harbin Institute of Technology, Harbin 150001, China; 2 Department of Materials Science and Engineering, Northwestern Polytechnical University, Xi’an 710072, China

**Keywords:** hybrid additive and subtractive manufacturing, electrical discharge machining, 316L, strength, surface roughness

## Abstract

In this study, a novel hybrid additive and subtractive manufacturing method using pulsed arc plasma (PAP-HASM) was developed to better integrate additive and subtractive processes. The PAP-HASM process is based on the flexible application of pulsed arc plasma. In this PAP-HASM method, wire arc additive manufacturing using pulsed arc plasma (PAP-WAAM) and dry electrical discharge machining (EDM) milling were used as additive and subtractive techniques, respectively; both are thermal machining processes based on pulsed arc plasma, and both are dry machining techniques requiring no working fluids. The PAP-HASM can be easily realized by only changing the pulsed power supply and tool electrodes. A key technological challenge is that the recast layer on the part surface after dry EDM milling may have a detrimental effect on the component fabricated by PAP-HASM. Here, the hybrid manufacturing method developed in this study was validated with commonly used 316L stainless steel. Preliminary experimental results showed that the PAP-HASM specimens exhibited excellent tensile properties, with an ultimate tensile strength of 539 ± 8 MPa and elongation of 46 ± 4%, which were comparable to the PAP-WAAM specimens. The recast layer on the surface after dry EDM milling has no significant detrimental effect on the mechanical properties of the parts fabricated by PAP-HASM. In addition, compared with components fabricated by PAP-WAAM, those fabricated by PAP-HASM showed significantly better surface roughness.

## 1. Introduction

Metal additive manufacturing is an advanced method enabling the fabrication of three-dimensional metallic parts by progressively adding materials in a layer-by-layer fashion [1], which allows the automatic production of complex shapes and multi-material structures without the need for other expensive tooling or mold [2,3]. Moreover, compared with conventional subtractive techniques, metal additive manufacturing offers significantly higher raw material utilization [4]; therefore, metal additive manufacturing has been widely used to manufacture high-performance parts in many fields, such as the aerospace industry [5], biomedical engineering [6] and the energy industry [7]. However, the full application of additively manufactured parts in different industries is limited by the low geometric accuracy [8], low surface quality [9] and material inhomogeneity [10]. Conventional subtractive manufacturing allows the achievement of high surface quality and geometric accuracy; however, it is difficult, even infeasible, to apply in manufacturing parts with complex geometric features [11]. Therefore, hybrid additive and subtractive manufacturing (HASM), which takes advantage of both processes and enables the machining of complex shaped parts with high geometric accuracy and surface quality [12,13], has attracted a great deal of attention.

Numerous researchers have focused on developing new HASM methods. Song et al. [14] proposed a hybrid approach called “3D welding and milling”, which hybridizes gas metal arc welding and milling. Li et al. [15] developed a piece of 6-axis hybrid additive-subtractive manufacturing equipment, equipped with multiple additives and subtractive changeable heads and an integrated manufacturing platform. Karunakaran et al. [16] developed a low-cost hybrid process for metallic parts, using an arc welding unit and milling machine; this process requires only low-cost retrofitting of existing machines without altering their original functionalities, which is conducive to the full application of HASM in different industries. Compared with arc-based additive manufacturing, laser-based additive manufacturing exhibits better accuracy and reliability. Kerschbaumer et al. [17] reported on the integration of selective laser cladding into commercial 5-axis machine tools. Liu et al. [18] developed a hybrid process system that alternates selective laser melting (SLM) and milling each few layers until the entire part is finished.

The developed hybrid processes, reported in relevant literature, have shown that HASM can be effective in manufacturing high-quality metal parts. However, current research on HASM has mainly focused on the hybridization of additive processes and conventional cutting manufacturing as a subtractive process [13]. To avoid the contamination of the additive process by residual cutting fluids, the use of such fluids must be avoided or minimized during the cutting process, which results in increased tool wear and reduced machining speeds and surface quality, particularly when machining difficult-to-machine materials, such as titanium, nickel and stainless steel alloys, constituting the main current application of metal additive manufacturing. At present, there is no agreement in academia and industry on how to solve the problem of residual cutting fluids, and only a few studies have been published [19,20]. Therefore, it is crucial to develop novel dry subtractive machining to achieve a more perfect integration of additive and subtractive processes [13].

Electrical discharge machining (EDM) has been widely used to manufacture metallic and non-metallic functional parts [21,22]. In particular, dry EDM milling [23] is an environmentally friendly EDM technology that uses gas as a dielectric medium instead of liquid. Pulse plasma channels at high frequencies (10^3^~10^6^ Hz) are created between the tool electrode and workpiece to remove unwanted materials by melting and evaporating [24]. High-pressure assist gas is jetted from the pipe tool electrode to remove the debris between the electrodes and cool the parts. A three-dimensional part can be manufactured layer by layer along a planned path with a pipe tool electrode [25]. Since dry EDM milling is a thermal erosion process, difficult-to-machine materials can be machined regardless of their hardness and strength. In addition, reaction forces formed in the EDM gap are insignificant; therefore, dry EDM milling can machine thin and complicated parts that are difficult to machine by cutting machining [24]. These advantages allow dry EDM milling to efficiently manufacture complex shaped components with high precision.

A wire arc additive manufacturing method using pulsed arc plasma (PAP-WAAM) has been proposed in our previous study to address the material processing challenges generated by high levels of heat input in WAAM [26]. However, the as-fabricated components are still difficult to use directly in the industry due to poor geometrical accuracy and surface quality.

Therefore, this study has developed a novel hybrid additive and subtractive manufacturing method using pulsed arc plasma (PAP-HASM) to better integrate the advantages of additive and subtractive processes. In this PAP-HASM method, PAP-WAAM and dry EDM were used as additive and subtractive techniques, respectively. Here, the basic process characteristics of PAP-HASM were first identified to show the advantages of this new hybrid manufacturing method. Then, the PAP-HASM method was used to fabricate 316L stainless steel (SS) parts whose characteristics, including the distribution of chemical composition, surface quality and mechanical properties, were thoroughly analyzed and discussed to assess the effectiveness of this hybrid process.

## 2. Materials and Methods

### 2.1. PAP-HASM

To achieve high-quality manufacturing of metal parts using WAAM, an innovative PAP-WAAM method has been developed in our previous study [26]. In this method, a pulsed arc plasma generated by the pulsed voltage was used as a heat source. By adjusting the relative positions of the tungsten electrode, filler wire and substrate, the arc plasma was ignited between the tungsten electrode and the filler wire, which increased the proportion of the discharge energy allocated to the filler wire during the discharge, thus reducing the overall heat input required for material deposition. During the discharge interval, the arc plasma was completely extinguished to increase the cooling rate of the materials. As a result of the lower heat input and higher cooling rate, the parts fabricated by PAP-WAAM exhibited finer geometric features than those fabricated by conventional WAAM. However, the as-fabricated components are still difficult to use directly in the industry due to poor geometrical accuracy and surface quality.

**Figure 1:**
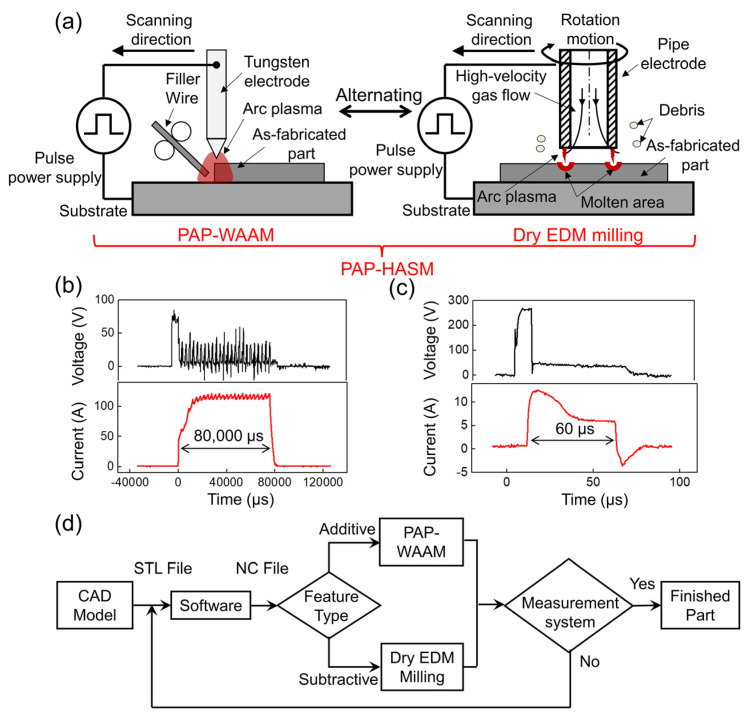
Hybrid additive and subtractive manufacturing method using pulsed arc plasma. (**a**) Schematic diagram; (**b**) measured voltage and current waveforms during PAP−WAAM; (**c**) measured voltage and current waveforms during dry EDM milling; (**d**) flow chart.

The PAP-HASM process is based on the flexible application of pulsed arc plasma. Figure 1a shows the schematic diagram of PAP-HASM. PAP-WAAM and dry EDM milling is performed alternately. Firstly, the PAP-WAAM is performed. A tungsten rod with a tip is used as the tool electrode, and a long discharge duration (10~10^3^ ms) is set to allow sufficient heat energy for the arc plasma to melt the filler wire. The arc plasma is used to melt the metal wire and deposit parts in a layer-by-layer fashion. Argon is used throughout the process to prevent oxidation. Subsequently, the pulsed power supply is switched to perform dry EDM milling, wherein the tool electrode is converted to a pipe electrode, and the discharge duration is reduced for the discharge to occur at high frequencies (10^3^~10^6^ Hz). For each pulse, the materials at the discharge spot are removed through melting and evaporation due to the high energy density of the arc plasma. With the assistance of the tool electrode rotation, a high velocity gas jet from a pipe tool electrode flushes the debris away from the discharge gap. Under continuous discharge, the expected shape or profile can be obtained by the accumulation of the removed material. Dry EDM milling is performed to remove unwanted materials from the deposition process. Figure 1b shows the measured voltage and current waveforms during the PAP-WAAM. Figure 1c shows the measured voltage and current waveforms during the dry EDM milling.

Figure 1d shows the process flow of the PAP-HASM. First, the computer-aided design (CAD) model (STL file) of parts and the process parameters are imported into the PAP-HASM software system. The CAD model is sliced into layers. Then, both the PAP-WAAM deposition and dry EDM milling paths are automatically generated by the PAP-HASM software. A layer is fabricated by depositing a single pass side by side according to the scanning paths. Once one or several layers are additively manufactured, the PAP-WAAM process is suspended, and dry EDM milling of the internal and external surfaces starts to remove unwanted materials from the deposition process according to the numerical control (NC) code. PAP-WAAM and dry EDM milling operate alternately until the entire part is finished.

### 2.2. Experimental Procedures

#### 2.2.1. Material Fabrication

**Figure 2:**
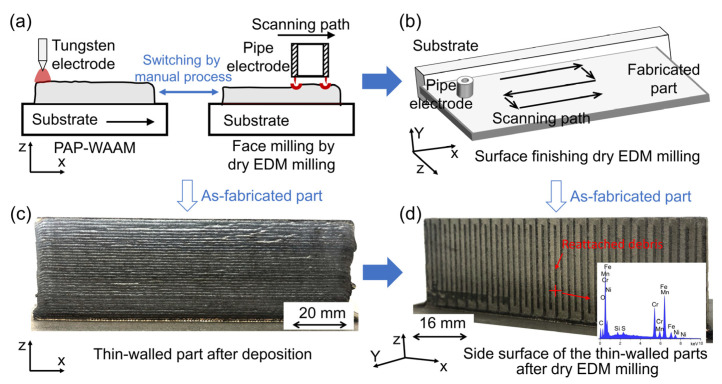
Schematic diagram of the PAP-HASM process (**a**,**b**) and as-fabricated parts (**c**,**d**) in this experiment.

To validate the developed PAP-HASM method, it was used to fabricate a 316L SS thin-walled structure with dimensions of 110 mm × 36 mm × 2 mm. Figure 2 shows the schematic diagram of the PAP-HASM process and as-fabricated parts in this experiment. First, the PAP-WAAM is performed (Figure 2a). The PAP-WAAM process was carried out using developed additive manufacturing devices consisting of a 200 A-rated pulse power supply (HB-J6; Hong Ben Shi Ye, Shanghai, China), a three-axis motion platform, a “cold” wire feeder. The experimental parameters of the PAP-WAAM are listed in Table 1. A commercial 316L SS wire with a diameter of 1.2 mm was melted using pulsed arc plasma and deposited on a rectangular 316L SS substrate with dimensions of 150 mm × 100 mm × 8 mm. Argon was used throughout the PAP-WAAM process to prevent oxidation. The chemical composition of the 316L SS wire is listed in Table 2. The layer thickness is approximately 1 mm. The PAP-WAAM is suspended each 10 layers, and dry EDM milling is performed to mill the top surface of the as-fabricated component while precisely controlling the thickness of the 10 layers at 9 mm (Figure 2a). The PAP-WAAM and dry EDM milling processes were manually switched in this study. Dry EDM milling was carried out using a precision EDM machine (DMK7140, Hanchuan Machine Tool Works, Hanzhong, China). Compressed air was used as an assist gas. Dry EDM milling parameters are listed in Table 3. The 36 mm-height component was fabricated using four additive and subtractive manufacturing cycles (Figure 2c). After finishing the sequence of deposition and face milling, surface finishing is performed by dry EDM milling to remove other unwanted materials, such as remaining stair steps on the side surface, and to ensure a 2 mm-width of the as-fabricated component (Figure 2b). Some debris reattach on the side of the part after dry EDM milling (Figure 2d). The mechanism underlying debris reattachment is described in detail in Section 3. The actual discharge voltage and current waveform during PAP-WAAM and dry EDM milling are presented in Figure 1b,c. In addition, to evaluate the mechanical properties of the PAP-HASM specimens, a 40-layer thin-walled structure was fabricated, as a counterpart, by PAP-WAAM under the same processing conditions (Table 1).

#### 2.2.2. Material Characterization

**Figure 3:**
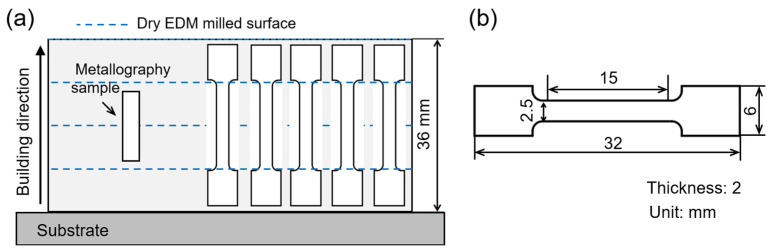
(**a**) The locations of the metallographic sample and tensile samples; (**b**) Tensile sample dimensions. Note: Both the tensile and metallurgical samples include dry EDM milled surfaces.

The samples required for both metallographic analyses and mechanical testing were cut off from as-built 316L SS parts using wire EDM. The locations of the metallographic and mechanical test samples are shown in Figure 3a. The metallographic specimens were first cold-mounted, ground and polished to mirror finish. Chemical composition analysis of the 316L SS part fabricated by PAP-HASM was performed by scanning electron microscopy (SEM) equipped with energy dispersive spectroscopy (EDS) (SU8000; Hitachi, Tokyo, Japan). In addition, the samples after polishing were electrochemically etched in a 10% oxalic acid solution in order to observe microstructure. The dimension of the tensile test samples is shown in Figure 3b. Tensile measurements were performed using a universal testing machine (AGXplus; Shimadzu, Kyoto, Japan) with a constant loading rate of 2 mm/min. The fracture surfaces and corresponding features of the tested samples were observed by SEM (Pharos G1; Phenom World, Eindhoven, The Netherlands). The Archimedes method was used to measure the relative density of as-fabricated specimens, where three specimens of the same type were measured to obtain an average value. The surface morphology and surface roughness of the as-fabricated parts were observed and measured using a super depth of field microscope (VHX-1000; Keyence, Osaka, Japan) and white light microscope (NewView 8200; Zygo, Middlefield, CT, USA), respectively.

## 3. Results and Discussion

**Figure 4:**
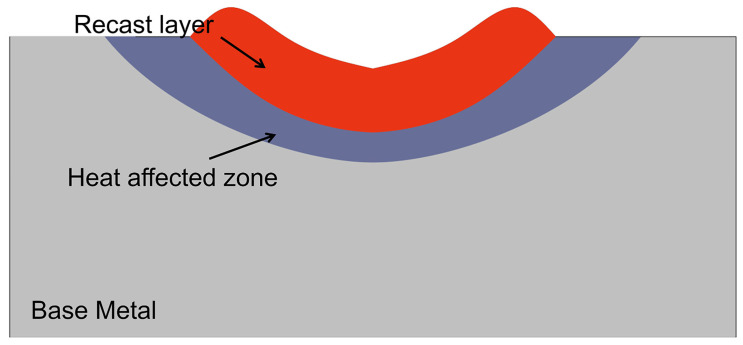
Schematic diagram of the EDM crater.

**Figure 5:**
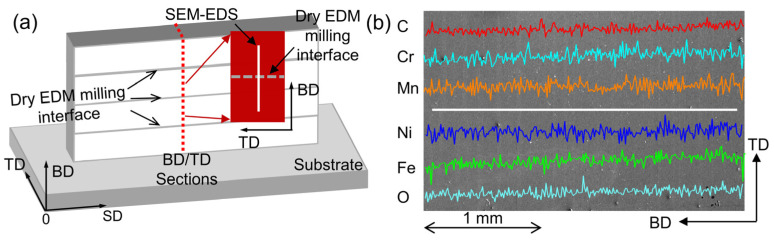
SEM-EDS line analysis of part fabricated by PAP-HASM: (**a**) Location of the SEM-EDS line spectrum; (**b**) results of the SEM-EDS line spectrum.

**Figure 6:**
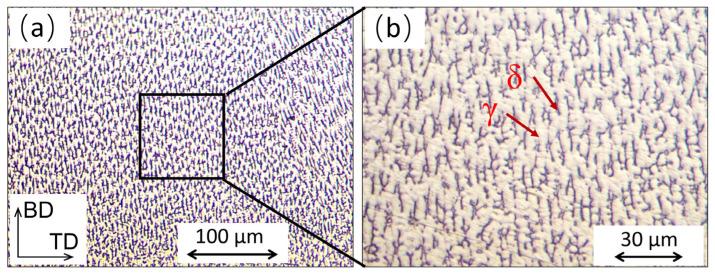
Representative SEM images of the 316L samples fabricated by PAP-WAAM.

Based on our understanding of the PAP-HASM process, there is a technological challenge that needs to be considered to realize this hybrid manufacturing process. Figure 4 shows the schematic diagram of the dry EDM crater. Dry EDM milling is a thermal erosion process wherein the unwanted materials are removed by melting and evaporation [24]. However, the unremoved materials inside the molten pool are resolidified on the surface of the part to form a recast layer (Figure 4). As a result, after dry EDM, the surface layer of the part usually experiences oxidation when air is used as a flushing gas. In PAP-HASM, owing to the alternation of PAP-WAAM and Dry EDM milling, the oxidation of the part surface after dry EDM milling may have a detrimental effect on the as-fabricated component. To investigate the effect of the recast layer on the as-fabricated component, the distribution of the chemical composition along the build direction was analyzed by SEM-EDS. Figure 5a shows the location of the SEM-EDS line spectrum. The measurement location crosses the dry EDM milling interface. Figure 5b shows the results of the SEM-EDS line spectrum crossing the dry EDM milling interface. The distribution of the chemical composition crossing the dry EDM milling interface is relatively uniform, without significant variations in the carbon and oxygen content. This indicates that the dry EDM milling did not significantly affect the chemical composition of the 316L SS parts fabricated by PAP-HASM. This phenomenon may be due to the recast layer on the part surface being very thin due to the large amount of molten material removed by the assist gas [24,25]. Figure 6 shows the microstructure of 316L samples fabricated by PAP-WAAM. Some gray δ-ferrite is present in the white γ-austenite matrix. The grains grow almost along the direction of the build, which agrees with previous reports [27].

**Figure 7:**
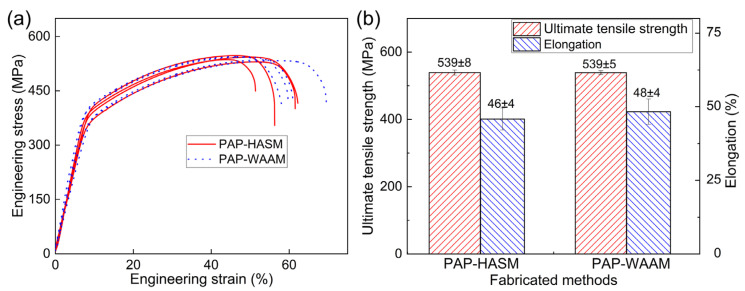
The mechanical properties of 316 L samples fabricated by PAP-HASM and PAP-WAAM. (**a**) Engineering stress–strain curves; (**b**) ultimate tensile strength and elongation.

Figure 7a shows the engineering stress-strain curves of the tensile specimens fabricated by PAP-HASM and PAP-WAAM. The corresponding ultimate tensile strength and elongation are shown in Figure 7b. The mean ultimate tensile strength and elongation of the PAP-HASM specimens are 539 ± 8 MPa and 46 ± 4%, respectively. The PAP-WAAM specimens exhibit similar ultimate tensile strength and elongation, with a mean ultimate tensile strength of 539 ± 5 MPa and a mean elongation of 48 ± 4%. The PAP-HASM components exhibit excellent tensile properties along the vertical direction, which are comparable to those of the PAP-WAAM components. This indicates that dry EDM milling did not cause adverse effects on the mechanical properties of the PAP-HASM components. Similarly, this phenomenon may be due to the recast layer on the part surface being very thin due to the large amount of molten material removed by the assist gas [24,25]. Dry EDM milling is performed to mill the top surface of the as-fabricated component with a height of approximately 9 mm. The recast layer has a very small proportion of the volume of the deposited structure. In addition, the recast layer is not 100% oxygen, and it is not possible to dilute all the material in the recast layer into the melt pool as some percentage of the material may vaporize. Thus, potential dissolution of the recast layer during PAP-HASM did not have a significant adverse effect on the mechanical properties of the as-fabricated parts. Similar analyses have been reported in the published literature [28].

**Figure 8:**
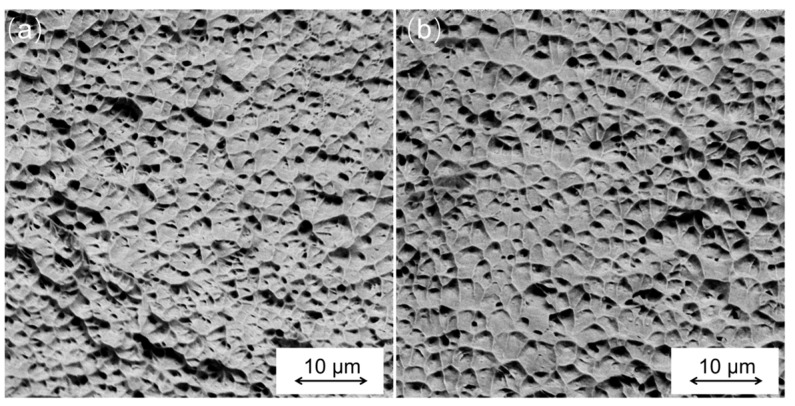
Representative SEM images of the fracture surface morphologies of the components fabricated by: (**a**) PAP-HASM; (**b**) PAP-WAAM.

**Figure 9:**
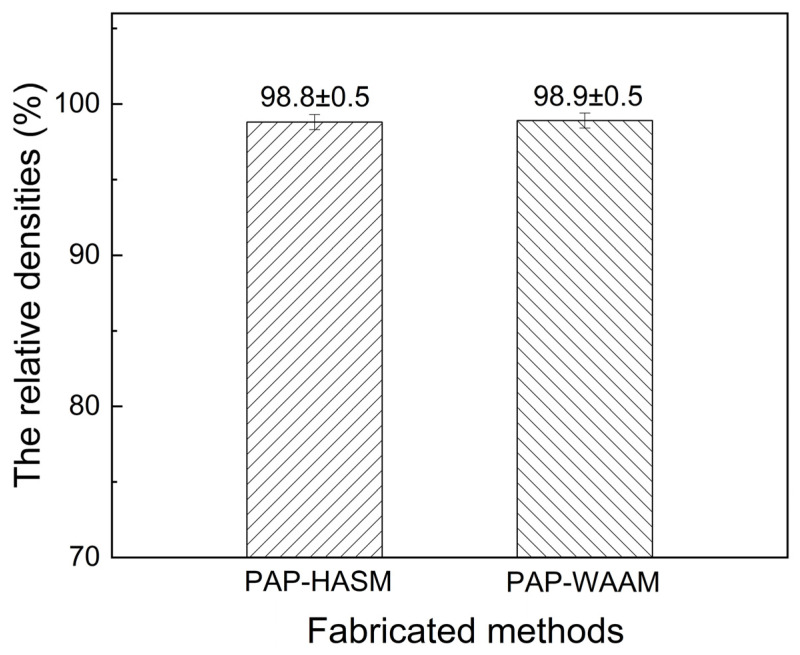
Relative density of specimens measured by the Archimedes method.

Figure 8 shows a representative fracture surface of the parts fabricated by PAP-HASM and PAP-WAAM. A number of dimple-like structures are observed on the fracture surfaces of both the PAP-HASM and PAP-WAAM specimens, which means that the specimens exhibit ductile fracture. The dimple morphologies can reflect the mechanical properties of the specimen. Evidently, the dimple morphology is almost identical on the fracture surface of both the PAP-HASM and PAP-WAAM components, indicating similar mechanical properties. The tensile properties shown in Figure 7 are consistent with the observed fracture surfaces, as shown in Figure 8. In addition, the relative density of the specimens fabricated by PAP-HASM and PAP-WAAM is shown in Figure 9. PAP-HASM and PAP-WAAM can produce nearly full dense 316 L components with a relative density ≥98.8%.

**Figure 10:**
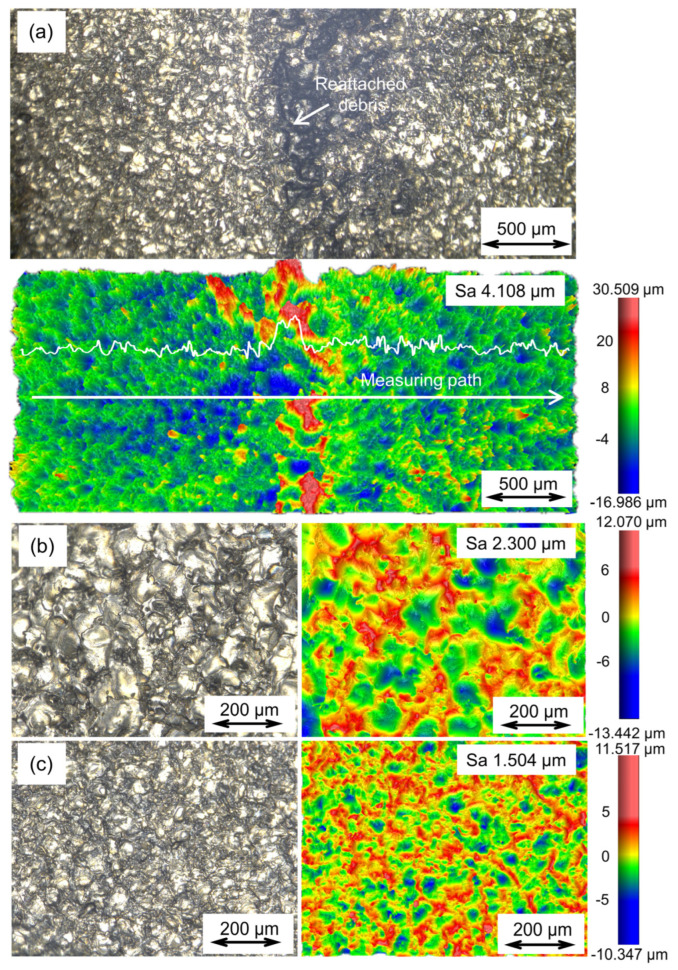
Surface morphology and surface roughness of as−fabricated parts under different conditions: (**a**) the side of the component is milled with the 1st finishing parameters; (**b**) the top surface of the component is milled with the 1st finishing parameters; (**c**) the top surface of the component is milled with the 3rd finishing parameters. Note: The surface morphology and surface roughness of the as−fabricated parts were observed and measured using a super depth of field microscope and white light microscope, respectively.

As shown in Figure 2d, some debris are reattached on the side of the part after dry EDM milling with the 1st finishing parameters. The debris is uniformly distributed along the scanning path of the dry EDM milling; this phenomenon may be due to some molten material that was expelled from the molten pool and which was not completely cooled when it reached the surface of the workpiece, forming debris reattached to the machined surface. The reattached debris is uniformly produced along the scanning path as the electrode moves. The surface morphology of the corresponding area is shown in Figure 10a. The reattached debris exhibits a black color. A relatively convex morphology can be observed in the area of the reattached debris, which leads to a reduction in the surface quality. The surface arithmetic means deviation (Sa) of the area where the reattached debris is located is 4.108 μm.

Interestingly, after dry EDM milling under the same processing parameters, the component exhibits a clean top surface without any reattached debris (Figure 10b). Much finer surface quality was observed with an Sa of 2.300 μm. This may be due to the width of the as-fabricated walls being smaller than the outer diameter of the tool electrode when machining on the top surface, providing higher efficiency in removing discharge debris with high-pressure assist gas. The discharge debris can be removed directly from the workpiece. The surface roughness of the part after dry EDM is determined by the discharge energy. The lower the discharge energy results in better surface roughness. Figure 10c shows the top surface of the component after dry EDM milling with the 3rd finishing parameters. Dry EDM milling parameters are shown in Table 3. The surface roughness is further reduced to an Sa of 1.504 um. The surface roughness can be further reduced by reducing the discharge energy. It is worth emphasizing that the filler wire was melted and deposited on the top surface without reattached debris during the PAP-HASM in this study. Thus, the dry EDM milling did not have a negative impact on the mechanical properties of the PAP-HASM component, as shown in Figure 7. At present, PAP-HASM is only suitable for the fabrication of thin-walled structures to avoid the effects of reattached debris. Further research is planned to address the issue of the reattached debris by optimizing the processing strategy.

**Figure 11:**
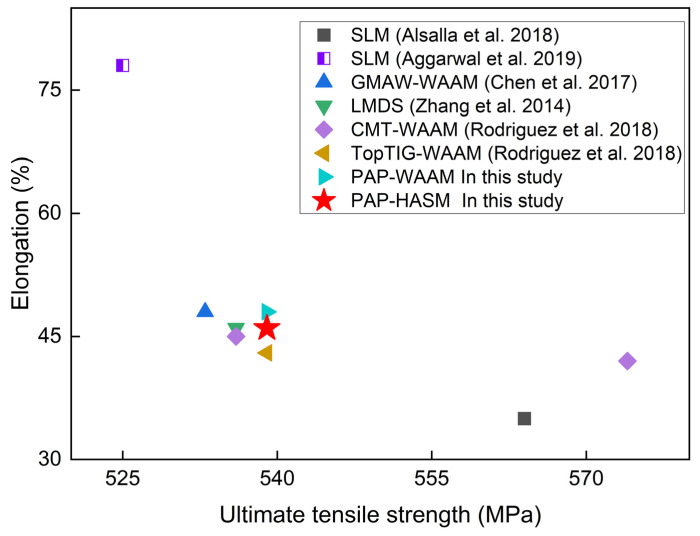
Comparison of mechanical properties between the present work and other relevant references [29,30,31,32,33]. Note: Gas metal arc welding (GMAW); laser metal deposition shaping (LMDS); cold metal transfer (CMT).

**Figure 12:**
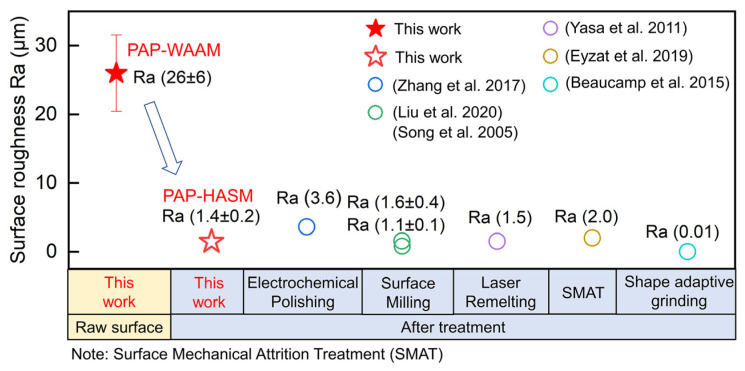
Comparison of surface roughness between the present work and other relevant references [14,18,34,35,36,37].

The experimental results obtained in this study are compared with those published in the relevant references. Figure 11 shows the comparison of mechanical properties, and Figure 12 shows the comparison of surface roughness. Because the mechanical properties of as-fabricated parts are determined by the forming methods and numerous processing parameters, a wide range of mechanical properties have been published in the references, with ultimate tensile strength ranging from 525 to 574 MPa and elongation ranging from 35% to 78%. The mechanical properties of components fabricated by PAP-HASM also fall in this range. This indicates that the PAP-HASM developed in this study can be used as an effective method to fabricate nearly full-dense metal parts with excellent mechanical properties. Figure 12 shows that the surface roughness of as-fabricated parts can be reduced to a Ra of 1.4 ± 0.2 μm after dry EDM milling with the 3rd finishing parameter. The surface roughness can be further reduced by reducing the discharge energy. This value is comparable to that resulting from commonly used post-treatment techniques, such as surface milling, laser remelting and surface mechanical attrition treatment, which is better than electrochemical polishing but inferior to shape adaptive grinding, indicating that the surface quality of PAP-HASM components can meet the demands of most engineering applications.

## 4. Conclusions

In this study, a novel PAP-HASM method, using PAP-WAAM and dry EDM as additive and subtractive techniques, respectively, is developed. Both additive and subtractive techniques are thermal machining processes based on pulsed arc plasma, which contributes to a better integration of the addition and subtraction processes. The hybrid manufacturing method developed in this study was validated with commonly used 316L stainless steel. PAP-HASM components exhibit excellent mechanical properties and surface roughness, with a mean ultimate tensile strength of 539 ± 8 MPa, mean elongation of 46 ± 4% and Sa of 1.504. The recast layer on the surface after dry EDM milling has no significant detrimental effect on the mechanical properties of the parts fabricated by PAP-HASM. This indicates that PAP-HASM is a potentially useful method for forming high-quality metal parts at a low cost.

After dry EDM milling, an amount of debris is distributed uniformly along the scanning path of the tool electrode on the side surface of the as-fabricated part. This phenomenon may be due to some molten material expelled from the molten pool and not completely cooled when reaching the surface of the workpiece, which forms debris reattached to the machined surface. The reattached debris has a detrimental effect on surface quality. Further research is planned to address the issue of the reattached debris by optimizing the processing strategy.

## Figures and Tables

**Table 1:** Additive manufacturing parameters.

Process Parameters	Details
Methods of AM	PAP-WAAM
Mean discharge current (A)	109.9
Mean discharge voltage (V)	13.4
Discharge duration (ms)	80
Discharge interval (ms)	300
Travel speed (mm/min)	150
Wire feed speed (mm/min)	37
Distance between electrode and workpiece (mm)	2.3
Shield gas flow rate (argon) (L/min)	20

**Table 2:** Chemical composition of 316L SS in this study (weight %).

Elements	C	Cr	Ni	Mn	Si	Mo	S	P	Fe
316L	0.015	19.27	12.47	1.61	0.38	2.21	0.009	0.018	Bal.

**Table 3:** Dry EDM milling parameters.

	Roughing	1st-Finishing	2nd-Finishing	3nd-Finishing
Discharge current	16 A	16 A	11 A	6.8 A
Discharge duration	60 μs	10 μs	5 μs	2 μs
Discharge interval	60 μs	10 μs	6 μs	6 μs
Depth of cut	100 μm	40 μm	10 μm	5 μm
Open voltage	270 V			
Polarity	Workpiece (+)			
Tool electrode	Cu Outer diameter: 4 mm Inner diameter: 3 mm			
Rotating speed of tool electrode	40 rpm			
Assist gas	Compressed air (0.5 MPa)			

## Data Availability

Not applicable.
